# Intensive care unit length of stay beyond the first week and 1-year mortality - dutch single centre study in unselected critically ill patients describing long-term survival according to length of stay in conjunction with age

**DOI:** 10.1186/2197-425X-3-S1-A149

**Published:** 2015-10-01

**Authors:** IW Soliman, DW de Lange, LM Peelen, W Pasma, JJM van Delden, D van Dijk

**Affiliations:** University Medical Center Utrecht, Department of Intensive Care, Utrecht, Netherlands; Julius Center for Health Sciences and Primary Care University Medical Center Utrecht, Department of Epidemiology, Utrecht, Netherlands; Julius Center for Health Sciences and Primary Care University Medical Center Utrecht, Department of Medical Humanities, Utrecht, Netherlands

## Introduction

Increasing age and prolonged intensive care unit (ICU) length of stay (LoS) are both associated with in-hospital mortality. Especially older patients with a prolonged ICU stay are often considered to be at high risk for mortality.[1] However the effect of a prolonged ICU LoS in conjunction with age on long-term survival remained to be specified.

## Objectives

We aimed to report one-year survival of ICU patients across different age strata and different LoS strata.

## Methods

All consecutive patients admitted to the ICU of the University Medical Centre Utrecht in the Netherlands between July 2009 and April 2013 were included in the study. For patients with multiple ICU admissions we only included their first admission. Data on patient characteristics were prospectively gathered according to APACHE IV definitions.[2] Survival was tracked using the municipal registry, until one year after ICU admission. Patients were grouped according to age and according to LoS. Note that the LoS groups were not mutually exclusive. The ethics committee of the University Medical Centre Utrecht approved the study and gave a waiver of informed consent (protocol number 10/006).

## Results

**Figure 1:**
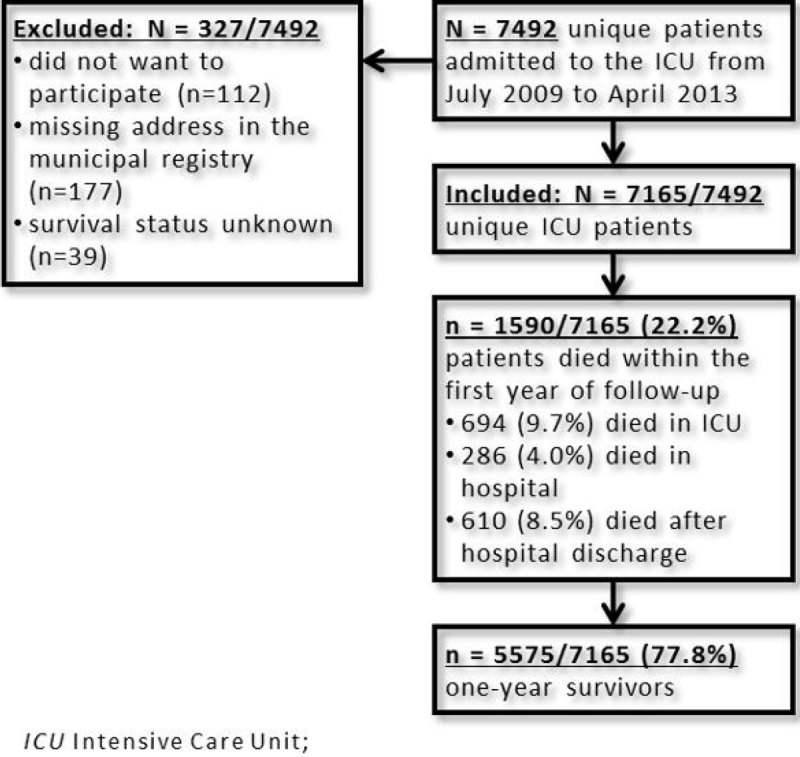
**Flowchart of inclusion and overall survival.**

**Figure 2:**
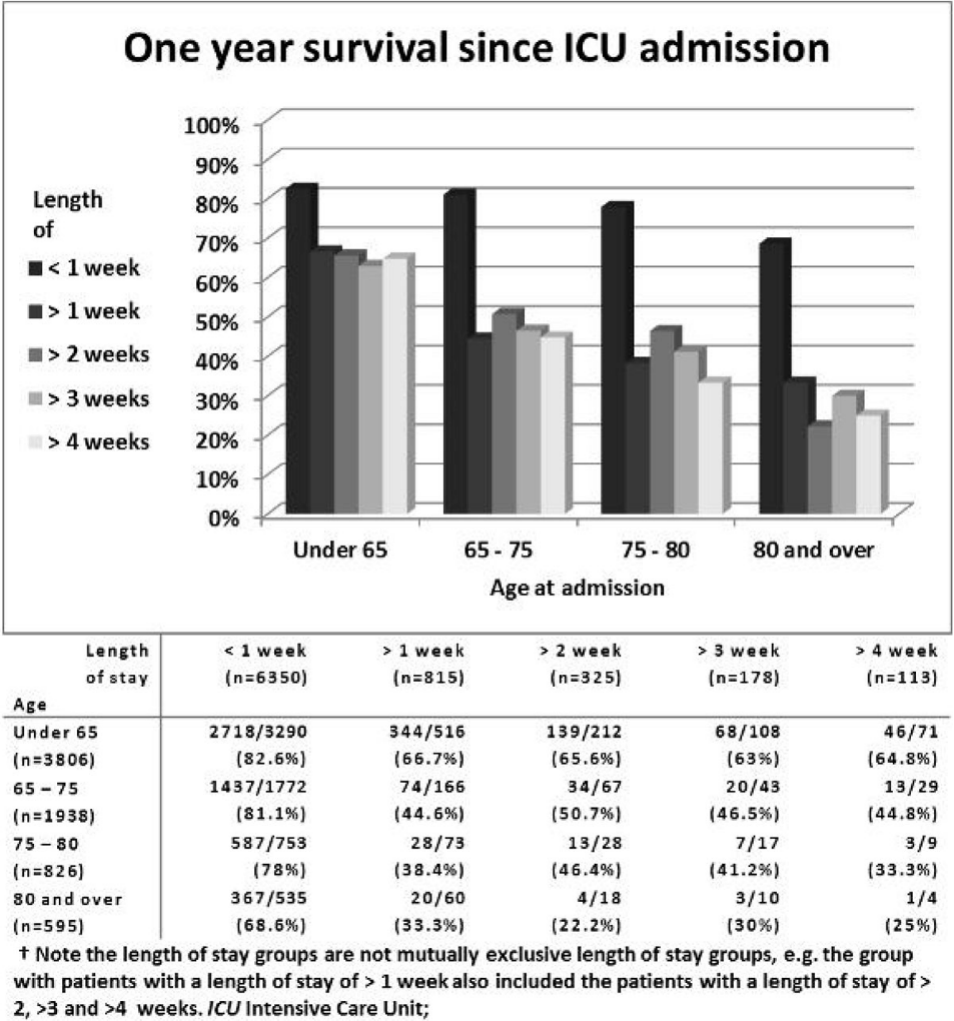
**One year survival since ICU admission.**

Inclusion and overall survival were shown in figure 1. In total, 7165 unique patients were included. Baseline characteristics are shown in table 1. One year survival varied markedly, from 82.6% in patients < 65 years with ICU LoS < 1 week, to 22.2% in patients > 80 years with ICU LoS > 2 weeks (figure 2). In all LoS strata, increasing age was associated with a decreasing one year survival. There was also a distinct drop in one year survival when ICU LoS was more than 1 week, in every age category. However, after the first week in the ICU, one year survival stayed nearly consistent in patients of all age categories, irrespective of ICU LoS.

**Table 1:** Baseline characteristics.

		Age at admission			
	Total population	Under 65	65-75	75-80	80 and over
**N**	7165	3806	1938	826	595
**Age (years)**	64 (52-73)	53 (43-60)	70 (67-72)	77 (76-79)	83 (81-85)
**Gender (male)**	4514 (63%)	2424 (63.7%)	1254 (64.7%)	505 (61.1%)	331 (55.6%)
**ICU length of stay (days)**	1 (1-3)	1 (1-4)	1 (1-2)	1 (1-2)	1 (1-3)
**Elective admission**	3908 (54.5%)	1764 (46.3%)	1265 (65.3%)	547 (66.2%)	332 (55.8%)
**APACHE IV score**	46 (33-66)	40 (27-62)	49 (38-65)	52 (41-69)	58 (46-77)
Total maximum SOFA score*	5 (2-9)	5 (2-9)	5 (2-8)	5 (3-8)	6 (3-10)

Continuous variables are presented as median (interquartile range), categorical variables as n (percentage); * Sum of highest SOFA component scores during admission. ICU Intensive Care Unit; LoS Length of Stay; APACHE IV Acute Physiology And Chronic Health Evaluation fourth edition; SOFA Sequential Organ Failure Assessment;

## Conclusions

In ICU patients, increasing age was associated higher one year mortality. However, once the patient has survived more than 2, 3 and 4 weeks of ICU stay respectively, his or her 1-year survival is comparable to patients who have been at the ICU for only more than one week.

## Grant Acknowledgement

This study was supported by the NutsOhra Foundation, project nr 1404-013.
